# 3,5-Diphenyl-1,2,4-dithia­zolium tetra­bromidoferrate(III)

**DOI:** 10.1107/S1600536813000275

**Published:** 2013-01-12

**Authors:** Ibukun O. Shotonwa, René T. Boeré

**Affiliations:** a Department of Chemistry and Biochemistry, University of Lethbridge, Lethbridge, Alberta, Canada T1K 3M4

## Abstract

The cation of the title salt, (C_14_H_10_NS_2_)[FeBr_4_], contains a flat central NC_2_S_2_ ring (r.m.s. deviation = 0.005 Å), with two attached phenyl rings that are almost coplanar [the dihedral angles between the mean planes are 2.4 (1) and 7.7 (1)° for the two phenyl rings]. The [FeBr_4_]^−^ anion makes short Br⋯S contacts [Br⋯S = 3.4819 (8), 3.6327 (9) and 3.5925 (9) Å] and also bridges by way of short contacts to ring H atoms of a second cation held parallel to the first by π-stacking, with a separation between the mean 1,2,4-dithia­zolium rings of 3.409 Å. The closest contacts are between a phenyl ring centroid of one cation and the *ipso* C atom of the phenyl ring of another cation, for which the distance is 3.489 Å. The discrete dimers are linked laterally by further Br⋯H short contacts, resulting in double sheets located parallel to the *b* axis and to the bis­ector of *a* and *c*.

## Related literature

For synthesis details, see: Corsaro *et al.* (1984 ▶); Liebscher & Hartmann (1977 ▶). For related structures, see: Clegg *et al.* (1996 ▶); Neels *et al.* (1986 ▶). For a description of the Cambridge Structural Database, see: Allen (2002 ▶). For bond lengths in tetrabromidoferrate complexes, see: Maithufi & Otto (2011 ▶); Bhattacharya & Sarkar (2010 ▶). For short-contact distances between stacked aryl rings, see: Martinez & Iverson (2012 ▶); McGaughey *et al.* (1998 ▶).
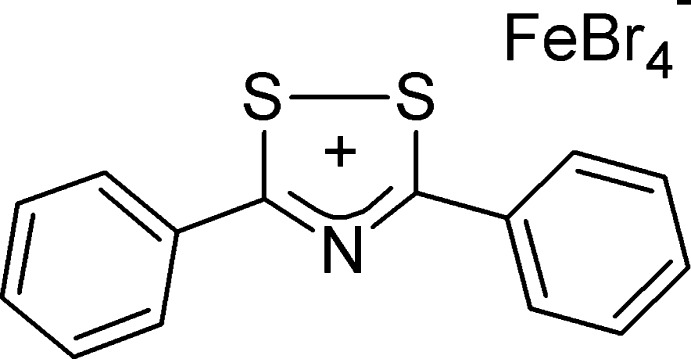


## Experimental

### 

#### Crystal data

(C_14_H_10_NS_2_)[FeBr_4_]
*M* *_r_* = 631.84
Monoclinic, 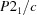

*a* = 10.6492 (5) Å
*b* = 11.4005 (6) Å
*c* = 15.8046 (8) Å
β = 96.482 (1)°
*V* = 1906.51 (17) Å^3^
*Z* = 4
Mo *K*α radiation
μ = 9.39 mm^−1^
*T* = 173 K
0.22 × 0.13 × 0.07 mm

#### Data collection

Bruker APEXII CCD area-detector diffractometer
Absorption correction: multi-scan (*SADABS*; Bruker, 2008 ▶) *T*_min_ = 0.556, *T*_max_ = 0.746
27655 measured reflections
4426 independent reflections
3497 reflections with *I* > 2σ(*I*)
*R*_int_ = 0.042

#### Refinement

*R*[*F* ^2^ > 2σ(*F* ^2^)] = 0.027
*wR*(*F* ^2^) = 0.050
*S* = 1.02
4426 reflections
199 parameters
H-atom parameters constrained
Δρ_max_ = 0.63 e Å^−3^
Δρ_min_ = −0.45 e Å^−3^

### 

Data collection: *APEX2* (Bruker, 2008 ▶); cell refinement: *SAINT-Plus* (Bruker, 2008 ▶); data reduction: *SAINT-Plus*; program(s) used to solve structure: *SHELXS97* (Sheldrick, 2008 ▶); program(s) used to refine structure: *SHELXTL* (Sheldrick, 2008 ▶); molecular graphics: Mercury (Macrae *et al.*, 2008 ▶); software used to prepare material for publication: *publCIF* (Westrip, 2010 ▶).

## Supplementary Material

Crystal structure: contains datablock(s) I, global. DOI: 10.1107/S1600536813000275/qm2091sup1.cif

Structure factors: contains datablock(s) I. DOI: 10.1107/S1600536813000275/qm2091Isup2.hkl

Additional supplementary materials:  crystallographic information; 3D view; checkCIF report

## Figures and Tables

**Fig. 1.**
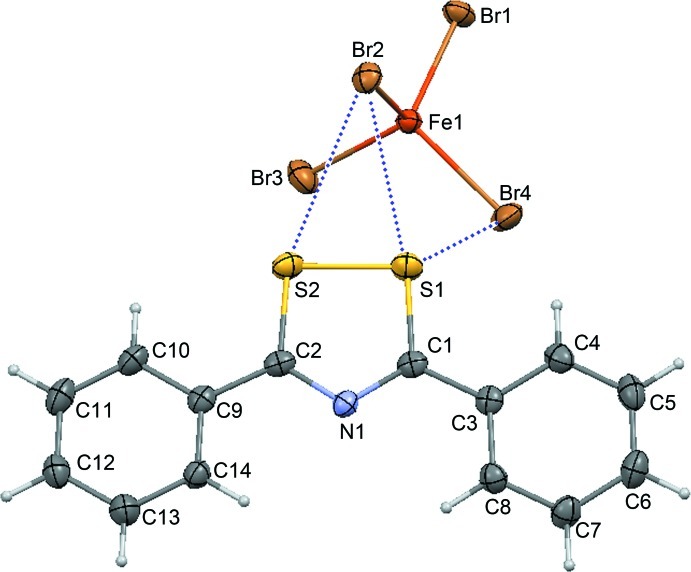
A view of (I) plotted with displacement ellipsoids drawn at the 40% probability level. H atoms are shown as spheres of arbitrary radius. Dotted lines indicate the short cation–anion contacts Br2···S1 = 3.4819 (8), Br2···S2 = 3.6327 (9) and Br4···S1 = 3.5925 (9) Å.

**Fig. 2.**
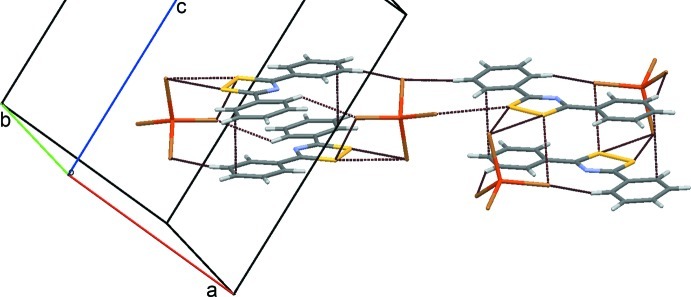
Short off-center parallel stacking of electron-rich aromatic rings leads to sheets of double layers parallel to b and bisecting the a and c axes; [FeBr4]- anions bridge between the upper and lower double-layer components.

## supplementary crystallographic information

### Comment

The structure of a cation–anion pair in (I) as found in the crystal lattice is shown in Fig. 1, wherein the short contacts from the anion Br atoms to the S atoms of the cation are highlighted. An extended diagram showing the dimerization of two rings through short inter-ring interactions and their lateral extensions into planes *via* short contacts between ring S and H atoms with the [FeBr_4_]^-^ anions is shown in Fig. 2. The separation between the approximately planar dimer components of 3.409 Å corresponds to typical minimum distances for off-center parallel stacking of electron-rich aromatic rings (Martinez & Iverson, 2012; McGaughey *et al.*, 1998).

Only two previous structures (II) and (III) are reported in the literature for 3,5-diaryl-1,2,4-dithiazolium ring compounds. For (II), the same 3,5-diphenyl-1,2,4-dithiazolium cation crystallizes with a large azathiophosphine cage anion, 1,3,5,7-tetrathioxo-10-aza-2,4,6,8,9-pentathia-1λ^5^,3λ^5^,5λ^5^,7λ^5^-tetraphosphatricyclo(3.3.1.1^3,7^)decanide [Cambridge Structural Database (Allen, 2002) refcode FARXIF (Neels *et al.*, 1986)]. For (III), the anion is the more conventional AsF_6_^-^, but the substituents are 2-methylbenzene rings [refcode TEHRED (Clegg *et al.*, 1996)]. The S—S bond in (I) at 2.019 (1) Å is significantly longer than that in (III) at 2.004 (1) Å, but is within the e.s.d. range of that found in (II) at 2.008 (5) Å. A possible reason for the longer bond in (I) is the presence of stronger sulfur-halogen non-bonded contact interactions than occur for AsF_6_^-^. The packing arrangements in (II) and (III) do not involve cation ring dimerization as observed in (I).

The bond distances in the [FeBr_4_]^-^ anion are entirely normal for high spin Fe^III^ (Fe—Br (av) = 2.332 (3) Å). For example, in a recent structure by Bhattacharya & Sarkar (2010) [refcode AKAZOC], Fe—Br (av) = 2.332 (7) Å. Distances in FeBr_4_^2-^ (*i.e.* an Fe^II^ anion) are as much as 6% longer (Maithufi & Otto, 2011).

### Experimental

Crystals of (I) were obtained as a minor component during the synthesis of 3,5-diphenyl-1,2,4-dithiazolium perchlorate from *N,N*-dimorpholino-thiobenzamide bromine adduct and unsubstituted thiobenzamide in chloroform using the method of Corsaro *et al.* (1984). The intermediates are taken up in 6 N HClO_4_ which is supposed to afford the perchlorate salt of the dithiazolium (Liebscher & Hartmann, 1977). The source of the iron is presumed to be rust that was digested by the perchloric acid, because the ferric aqua ion would be ripe for ligation by strongly nucleophilic bromide ions present as a byproduct from the previous step. Crystals of (I) form as well shaped red–orange prisms amongst the thin yellow plates of perchlorate salt, m.p. 169.3–171.1°C.

### Refinement

All H atoms were located on a difference map, but for purposes of refinement are treated as riding on their attached aromatic C atoms with C—H = 0.95 Å and *U*_iso_(H) = 1.2*U*_eq_(C).

### Crystal data

**Table:**

(C_14_H_10_NS_2_)[FeBr_4_]	Standard setting
*M**_r_* = 631.84	*D*_x_ = 2.201 Mg m^−^^3^
Monoclinic, *P*2_1_/*c*	Melting point: 442.5 K
Hall symbol: -P 2ybc	Mo *K*α radiation, λ = 0.71073 Å
*a* = 10.6492 (5) Å	Cell parameters from 9924 reflections
*b* = 11.4005 (6) Å	θ = 2.2–27.5°
*c* = 15.8046 (8) Å	µ = 9.39 mm^−^^1^
β = 96.482 (1)°	*T* = 173 K
*V* = 1906.51 (17) Å^3^	Prism, red
*Z* = 4	0.22 × 0.13 × 0.07 mm
*F*(000) = 1196	

### Data collection

**Table:**

Bruker APEXII CCD area-detector diffractometer	4426 independent reflections
Radiation source: fine-focus sealed tube, Bruker D8	3497 reflections with *I* > 2σ(*I*)
Graphite monochromator	*R*_int_ = 0.042
Detector resolution: 66.06 pixels mm^-1^	θ_max_ = 27.7°, θ_min_ = 1.9°
φ and ω scans	*h* = −13→13
Absorption correction: multi-scan (*SADABS*; Bruker, 2008)	*k* = −14→14
*T*_min_ = 0.556, *T*_max_ = 0.746	*l* = −20→20
27655 measured reflections	

### Refinement

**Table:**

Refinement on *F*^2^	Primary atom site location: structure-invariant direct methods
Least-squares matrix: full	Secondary atom site location: difference Fourier map
*R*[*F*^2^ > 2σ(*F*^2^)] = 0.027	Hydrogen site location: difference Fourier map
*wR*(*F*^2^) = 0.050	H-atom parameters constrained
*S* = 1.02	*w* = 1/[σ^2^(*F*_o_^2^) + (0.0202*P*)^2^ + 0.6153*P*] where *P* = (*F*_o_^2^ + 2*F*_c_^2^)/3
4426 reflections	(Δ/σ)_max_ = 0.001
199 parameters	Δρ_max_ = 0.63 e Å^−^^3^
0 restraints	Δρ_min_ = −0.45 e Å^−^^3^

### Special details

**Table:**

Experimental. A crystal coated in Paratone (TM) oil was mounted on the end of a thin glass capillary and cooled in the gas stream of the diffractometer Kryoflex device.
Geometry. All e.s.d.'s (except the e.s.d. in the dihedral angle between two l.s. planes) are estimated using the full covariance matrix. The cell e.s.d.'s are taken into account individually in the estimation of e.s.d.'s in distances, angles and torsion angles; correlations between e.s.d.'s in cell parameters are only used when they are defined by crystal symmetry. An approximate (isotropic) treatment of cell e.s.d.'s is used for estimating e.s.d.'s involving l.s. planes.
Refinement. Refinement of *F*^2^ against ALL reflections. The weighted *R*-factor *wR* and goodness of fit *S* are based on *F*^2^, conventional *R*-factors *R* are based on *F*, with *F* set to zero for negative *F*^2^. The threshold expression of *F*^2^ > σ(*F*^2^) is used only for calculating *R*-factors(gt) *etc*. and is not relevant to the choice of reflections for refinement. *R*-factors based on *F*^2^ are statistically about twice as large as those based on *F*, and *R*-factors based on ALL data will be even larger.

### Fractional atomic coordinates and isotropic or equivalent isotropic displacement parameters (Å^2^)

**Table:**

	*x*	*y*	*z*	*U*_iso_*/*U*_eq_	
Br2	0.52392 (3)	0.78560 (3)	0.368206 (19)	0.03449 (8)	
S2	0.66283 (7)	0.51567 (7)	0.45795 (5)	0.03375 (18)	
S1	0.76220 (7)	0.65596 (7)	0.50609 (5)	0.03592 (18)	
N1	0.8648 (2)	0.4568 (2)	0.56025 (14)	0.0258 (5)	
C1	0.8760 (2)	0.5727 (2)	0.56450 (16)	0.0264 (6)	
C2	0.7649 (3)	0.4159 (3)	0.51171 (16)	0.0273 (6)	
C3	0.9797 (3)	0.6304 (2)	0.61626 (17)	0.0266 (6)	
C4	0.9917 (3)	0.7527 (3)	0.61675 (19)	0.0342 (7)	
H4	0.9323	0.7991	0.5821	0.041*	
C5	1.0893 (3)	0.8059 (3)	0.6672 (2)	0.0412 (8)	
H5	1.0965	0.8889	0.6681	0.049*	
C6	1.1764 (3)	0.7384 (3)	0.71628 (19)	0.0399 (8)	
H6	1.2439	0.7751	0.7509	0.048*	
C7	1.1665 (3)	0.6175 (3)	0.71570 (18)	0.0371 (7)	
H7	1.2274	0.5717	0.7497	0.045*	
C8	1.0687 (3)	0.5628 (3)	0.66606 (17)	0.0315 (7)	
H8	1.0621	0.4797	0.6658	0.038*	
C9	0.7397 (3)	0.2903 (2)	0.50317 (17)	0.0276 (6)	
C10	0.6429 (3)	0.2465 (3)	0.44497 (18)	0.0347 (7)	
H10	0.5910	0.2985	0.4094	0.042*	
C11	0.6230 (3)	0.1269 (3)	0.4395 (2)	0.0416 (8)	
H11	0.5574	0.0966	0.3998	0.050*	
C12	0.6980 (3)	0.0510 (3)	0.49137 (18)	0.0369 (7)	
H12	0.6839	−0.0312	0.4871	0.044*	
C13	0.7932 (3)	0.0946 (3)	0.5493 (2)	0.0379 (7)	
H13	0.8441	0.0423	0.5853	0.045*	
C14	0.8147 (3)	0.2137 (2)	0.55519 (18)	0.0311 (7)	
H14	0.8808	0.2434	0.5948	0.037*	
Fe1	0.67510 (4)	0.82072 (3)	0.27504 (2)	0.02488 (10)	
Br1	0.61558 (3)	0.98672 (3)	0.194423 (18)	0.03480 (8)	
Br3	0.68944 (3)	0.65879 (3)	0.18652 (2)	0.04436 (9)	
Br4	0.87224 (3)	0.85136 (3)	0.35206 (2)	0.04081 (9)	

### Atomic displacement parameters (Å^2^)

**Table:**

	*U*^11^	*U*^22^	*U*^33^	*U*^12^	*U*^13^	*U*^23^
Br2	0.02809 (16)	0.03810 (18)	0.03845 (17)	−0.00452 (13)	0.00891 (13)	0.00027 (13)
S2	0.0321 (4)	0.0389 (4)	0.0287 (4)	0.0031 (3)	−0.0038 (3)	0.0027 (3)
S1	0.0400 (4)	0.0333 (4)	0.0329 (4)	0.0022 (3)	−0.0029 (3)	0.0078 (3)
N1	0.0239 (12)	0.0289 (13)	0.0247 (12)	−0.0012 (10)	0.0031 (10)	−0.0007 (10)
C1	0.0246 (15)	0.0331 (17)	0.0227 (14)	0.0011 (12)	0.0078 (12)	0.0033 (12)
C2	0.0241 (15)	0.0370 (17)	0.0214 (14)	0.0034 (12)	0.0046 (12)	0.0039 (12)
C3	0.0276 (15)	0.0306 (16)	0.0228 (14)	−0.0027 (12)	0.0089 (12)	0.0015 (12)
C4	0.0327 (17)	0.0301 (17)	0.0413 (18)	0.0011 (13)	0.0104 (14)	0.0039 (14)
C5	0.0426 (19)	0.0318 (18)	0.051 (2)	−0.0102 (15)	0.0146 (16)	−0.0056 (15)
C6	0.0397 (19)	0.046 (2)	0.0337 (17)	−0.0143 (15)	0.0019 (14)	−0.0044 (15)
C7	0.0392 (18)	0.0401 (19)	0.0307 (17)	−0.0056 (14)	−0.0019 (14)	0.0038 (14)
C8	0.0362 (17)	0.0290 (16)	0.0294 (16)	−0.0023 (13)	0.0044 (13)	0.0021 (13)
C9	0.0258 (15)	0.0332 (16)	0.0246 (14)	−0.0017 (12)	0.0065 (12)	−0.0014 (12)
C10	0.0273 (16)	0.045 (2)	0.0303 (16)	−0.0031 (14)	−0.0017 (13)	−0.0023 (14)
C11	0.0351 (18)	0.053 (2)	0.0358 (18)	−0.0111 (16)	0.0003 (14)	−0.0103 (16)
C12	0.0405 (19)	0.0312 (17)	0.0397 (18)	−0.0052 (14)	0.0078 (15)	−0.0068 (14)
C13	0.0378 (18)	0.0344 (18)	0.0409 (18)	−0.0014 (14)	0.0019 (14)	−0.0007 (14)
C14	0.0286 (16)	0.0334 (17)	0.0303 (15)	−0.0026 (13)	−0.0010 (12)	−0.0026 (13)
Fe1	0.0231 (2)	0.0242 (2)	0.0271 (2)	0.00182 (16)	0.00160 (16)	0.00272 (16)
Br1	0.04259 (18)	0.02889 (16)	0.03186 (16)	0.00849 (13)	−0.00040 (13)	0.00606 (12)
Br3	0.0519 (2)	0.02974 (18)	0.0529 (2)	0.00340 (15)	0.01216 (16)	−0.00932 (15)
Br4	0.02328 (15)	0.0481 (2)	0.0491 (2)	−0.00342 (14)	−0.00431 (13)	0.01204 (15)

### Geometric parameters (Å, º)

**Table:**

Br2—Fe1	2.3357 (5)	C7—H7	0.9500
S2—C2	1.729 (3)	C8—H8	0.9500
S2—S1	2.0186 (11)	C9—C14	1.389 (4)
S1—C1	1.723 (3)	C9—C10	1.394 (4)
N1—C2	1.324 (3)	C10—C11	1.381 (4)
N1—C1	1.329 (3)	C10—H10	0.9500
C1—C3	1.455 (4)	C11—C12	1.382 (4)
C2—C9	1.460 (4)	C11—H11	0.9500
C3—C8	1.394 (4)	C12—C13	1.379 (4)
C3—C4	1.399 (4)	C12—H12	0.9500
C4—C5	1.377 (4)	C13—C14	1.379 (4)
C4—H4	0.9500	C13—H13	0.9500
C5—C6	1.375 (4)	C14—H14	0.9500
C5—H5	0.9500	Fe1—Br1	2.3291 (5)
C6—C7	1.382 (4)	Fe1—Br4	2.3311 (5)
C6—H6	0.9500	Fe1—Br3	2.3317 (5)
C7—C8	1.380 (4)		
			
C2—S2—S1	93.58 (10)	C3—C8—H8	120.2
C1—S1—S2	94.17 (10)	C14—C9—C10	119.9 (3)
C2—N1—C1	116.2 (2)	C14—C9—C2	118.3 (2)
N1—C1—C3	122.4 (2)	C10—C9—C2	121.8 (3)
N1—C1—S1	117.9 (2)	C11—C10—C9	119.4 (3)
C3—C1—S1	119.7 (2)	C11—C10—H10	120.3
N1—C2—C9	121.8 (2)	C9—C10—H10	120.3
N1—C2—S2	118.2 (2)	C10—C11—C12	120.5 (3)
C9—C2—S2	120.1 (2)	C10—C11—H11	119.8
C8—C3—C4	119.5 (3)	C12—C11—H11	119.8
C8—C3—C1	119.5 (3)	C13—C12—C11	120.0 (3)
C4—C3—C1	121.1 (3)	C13—C12—H12	120.0
C5—C4—C3	120.3 (3)	C11—C12—H12	120.0
C5—C4—H4	119.9	C14—C13—C12	120.3 (3)
C3—C4—H4	119.9	C14—C13—H13	119.9
C6—C5—C4	119.8 (3)	C12—C13—H13	119.9
C6—C5—H5	120.1	C13—C14—C9	119.9 (3)
C4—C5—H5	120.1	C13—C14—H14	120.0
C5—C6—C7	120.5 (3)	C9—C14—H14	120.0
C5—C6—H6	119.7	Br1—Fe1—Br4	109.714 (19)
C7—C6—H6	119.7	Br1—Fe1—Br3	110.324 (19)
C8—C7—C6	120.4 (3)	Br4—Fe1—Br3	108.411 (19)
C8—C7—H7	119.8	Br1—Fe1—Br2	108.648 (19)
C6—C7—H7	119.8	Br4—Fe1—Br2	109.921 (19)
C7—C8—C3	119.5 (3)	Br3—Fe1—Br2	109.817 (19)
C7—C8—H8	120.2		
